# Distraction by violation of sensory predictions: Functional distinction between deviant sounds and unexpected silences

**DOI:** 10.1371/journal.pone.0274188

**Published:** 2022-09-06

**Authors:** Fabrice B. R. Parmentier, Alicia Leiva, Pilar Andrés, Murray T. Maybery

**Affiliations:** 1 Department of Psychology & Research Institute of Health Sciences, Neuropsychology & Cognition Group, University of the Balearic Islands, Palma, Balearic Islands, Spain; 2 Balearic Islands Health Research Institute (IdISBa), Palma, Balearic Islands, Spain; 3 School of Psychological Science, University of Western Australia, Perth, Western Australia, Australia; 4 Department of Psychology, Universitat de Vic-Universitat Central de Catalunya, Catalunya, Spain; Rutgers The State University of New Jersey, UNITED STATES

## Abstract

It has been established that participants performing a continuous categorization task respond significantly slower following the presentation of unexpected, task-irrelevant, auditory stimuli, compared to a repetitive (standard) sound. Evidence indicates that such distraction emerges because of the violation of sensory predictions. This has typically been studied by measuring the impact of replacing the repeated sound by a different sound on rare and unpredictable trials. Here, we examine the impact of a different type of violation: the mere omission of the standard sound. Capitalizing upon the recent finding that deviant sounds exert distinct effects on response times as a function of whether participants produced or withheld a response on the previous trial, we present the results of an experiment seeking to disentangle two potential effects of sound omission: deviance distraction and the removal of an unspecific warning signal. The results indicate that deviant sound and the unexpected omission of the standard sound impact response times through, at least partially, distinct mechanisms. Deviant sounds affect performance by triggering the orienting of attention towards a new sensory input. Sound omissions, in contrast, appear to affect performance in part because responses no longer benefit from an unspecific warning signal to prepare for action.

## Introduction

The ability to focus on a task and filter out task-irrelevant stimuli is undeniably essential to efficient cognitive functioning [1–4]. Equally pivotal is change detection [5–9], which allows salient or unexpected stimuli to break through attention filters to capture our attention and, if need be, call for the modification of an ongoing action. However, in situations where attention grabbing stimuli turn out to be entirely irrelevant, the result is distraction. In the laboratory, such distraction can for example be observed when unexpected task-irrelevant auditory stimuli deviating from an otherwise repetitive or structured sequence impact performance in an ongoing unrelated task [7,10–17].

In the cross-modal oddball task, participants typically categorize visual digits while instructed to ignore the sound preceding each digit. One sound is used on the majority of trials (standard sound), while a different sound is used on rare and unpredictable trials (deviant sound). Such deviation triggers a well-established series of electrophysiological responses (mismatch negativity or MMN, a positive deflection or P3a, and the reorientation negativity or RON), thought to reflect, respectively, the violation of predictions, the involuntary orientation of attention toward the deviant stimulus, and the re-orientation toward the primary task [11,15,18–23]. Importantly, deviant sounds also yield a lengthening of response times in the primary task [7,12,13]. Since its initial report [7], behavioral deviance distraction has been reported in numerous studies using auditory-visual oddball tasks [12,20,24–26], but also tactile-visual [27,28], purely visual [29–32], or purely auditory [7,11,33–41] tasks.

Behavioral and electrophysiological studies have shown that deviance distraction originates from the violation of sensory predictions rather than from the deviant sound’s low probability *per se* [42–46], is associated with a transient inhibition of actions [47–50], and is unaffected by response predictability [51]. Violations of predictions can potentially also take another form, however: the omission of the predicted auditory stimulus. That is, the mere omission of a predicted sound might potentially produce functionally similar effects to that of the presentation of a deviant sound.

There has been no attempt to examine the impact of auditory stimulus omission on behavioral performance in oddball tasks, though one study [52] examined the effect of stimulus omission in the tactile modality. In that study, an unexpected sound presented in lieu of a standard vibration produced longer response times, an effect due to the omission of the standard vibration rather than to the presentation of the sound. The authors concluded that the mere omission of the standard tactile stimulus constitutes a violation of sensory predictions in the same way as a deviant stimulus.

In the electrophysiological literature, some studies have reported electrophysiological responses to omissions resembling those triggered by deviant sounds [e.g., 53–58]. However, the MMN response to tone omission appears limited to rapidly presented tones [59] and builds up slower than the MMN elicited by violations of stimulus repetitions [60]. Hence, omissions and deviant sounds produce a MMN response through distinct mechanisms [61].

Studying the impact of sound omission on behavioral performance in the cross-modal oddball task is not straightforward because, while standard sounds elicit sensory predictions, sounds in this paradigm also fulfil the function of unspecific warning signals [62–66] that facilitate action [67,68]. This is illustrated, for example, by the finding that responses are slower in a silent block of trials than following the standard sound [10,12]. This presents a methodological problem: omitting the standard sound constitutes a violation of sensory predictions but it also results in the removal of a warning signal, both factors expected to lengthen response times. Here, we propose a functional solution to this methodological conundrum by capitalizing on recent work showing that deviant sounds elicit distinct (in fact opposite) effects on response times depending on whether participants performed the primary task or inhibited actions on the previous trial. As described next, we will argue that, using this distinction, it is possible to disentangle the two effects.

Revisiting the claim that deviant sounds yield no behavioral distraction in cross-modal oddball tasks in which sounds are deprived of their warning value [69–73], Parmentier [67] showed that this finding was a *fata morgana* resulting from the indiscriminate analysis of performance irrespective of whether the previous trial required a response (Go trial) or not (NoGo trial). Taking into account this distinction, the data reveal in fact two opposite effects: deviant sounds lengthen response times following Go trials but speed responses up following NoGo trials. Parmentier [67] argued that deviant sounds capture attention as they violate sensory predictions, and that this, in turn, modulates action plans in the following way: Immediately after a Go trial, deviant trials bring a change of context that renders more difficult the repetition of the currently active action plan. Following a NoGo trial, the change of context brought by the deviant sound helps disengaging from the state of action inhibition, thereby facilitating the reactivation of the relevant task set and the production of responses. Hence, following a standard Go trial, the cognitive system is geared toward action and a deviant sound will slow responses because it triggers a temporary inhibition of action plans and an involuntary attention capture. In contrast, following a standard NoGo trial, a deviant sound disengages the cognitive system from the state of motor inhibition it was geared toward, thereby speeding up responses.

Though not immediately apparent, the finding described above holds a key to the question at stake in the present study because the contrasting effects of deviant sounds following Go and NoGo trials are functionally distinct from the sounds’ warning value. If the cognitive system processes the silence resulting from the omission of the standard sound in the same way as it processes a deviant sound, that is, as a discrete departure from sensory predictions, then it should act, and affect response times, in the same way as a deviant sound. More specifically, compared to standard sounds, silence and deviant sounds should yield slower response times following Go trials, and faster response times following NoGo trials. In contrast, if silence simply amounts to the absence of an unspecific warning signal, then it should slow response times across the board (no unspecific preparation for action), irrespective of whether the previous trial was Go or NoGo. These two hypotheses were contrasted for the first time in an experiment in which participants performed a cross-modal oddball task in which two deviations from the standard sound were introduced: deviant sounds and silence.

## Method

### Participants

Thirty-two (23 women) undergraduates from the University of the Balearic Islands took part in this experiment in exchange for a small honorarium. Participants were aged 18 to 23 (M = 19.03, SD = 1.24). All participants reported correct or corrected-to-normal vision and normal hearing.

An initial power analysis was carried out based on an estimate of the size of the effect of critical importance in our study: the difference in distraction (deviant/silence vs standard) between PostGo and PostNoGo trials. We revisited four existing data sets comparing deviant and standard trials in PostGo and PostNoGo trials and calculated this effect size: d_az_ = 1.009 [73], d_az_ = 1.531 [69], d_az_ = 1.920 [70], and d_az_ = 1.337 [67]. Based on an average d_az_ of 1.450, Type I error probability of .05, and a power of .95, the minimum sample size was 9 [calculated using GPower, 74]. Based on the smallest of the above effect sizes, it was 13. We used this estimate as a minimum sample size requirement, advertised the experiment and allowed all participants interested to take part.

### Stimuli, design, and procedure

Participants were presented with 1800 test trials each (5 blocks of 360). On half of the trials (GO trials) a visual digit (1–6) was presented and participants categorized it as odd or even using two arbitrary allocated keys (X & Z, counterbalanced across participants), which they pressed using the index and middle fingers of their dominant hand. On the other half of the trials (NoGo trials), the fixation cross appeared instead of a digit and participants withheld responding. The fixation cross was visible at the center of the screen throughout the task except when replaced by the presentation of the to-be-attended visual digit.

Each trial began with the presentation of a task-irrelevant sound (150 ms) or a similar period of silence. This event was followed, after one of three equi-probable temporal intervals (0, 100, 200 ms) by the presentation of a visual digit or its substitution by the fixation cross (with equal probabilities). The visual digit or the fixation cross appeared at the center of a square box (sustaining a viewing angle of approximately 2.6°) and remained visible for 150 ms. An interval of 950 ms followed, thereby affording participants a total response window of 1100 ms. At the end of this interval, the next trial began automatically.

Three sound conditions were compared: standard (80% of trials), deviant (10%) and silent (10%) conditions. The three sound files corresponding to these conditions were, respectively: a 600 Hz sinewave tone, a burst of white noise, and silence. These sound files had a duration of 150 ms. The standard and deviant sounds were normalized and their envelope edited to include 10ms rise and fall ramps. They were delivered binaurally through headphones at an intensity of approximately 75 db SPL.

The digits (1–6) were presented in a different pseudo-random order for each participant, conforming to the following rules: each digit was used equally often across all conditions (3 sounds x 3 auditory-visual stimuli intervals x presence/omission of the visual target) across each successive group of 180 trials, and deviant and silent trials were followed by at least one standard trial.

Participants were instructed to ignore the task-irrelevant sounds and to concentrate on the digit categorization task, responding to these as quickly as possible while trying to be as accurate as possible. In each block, test trials were prefixed by the presentation of 12 standard practice trials that served as practice and warmup trials and that were excluded from the data analysis. Participants were allowed to take a short break between blocks if they wished to. The experimental session took approximately an hour.

This study was carried out in accordance with the recommendations of American Psychological Association with written informed consent from all subjects. All subjects gave written informed consent in accordance with the Declaration of Helsinki. The protocol was approved by the Bioethical Committee of the University of the Balearic Islands.

## Results

The mean proportion of correct responses and the mean response times (RTs) for correct responses were analyzed as a function of (1) the type of trial (whether a given trial was preceded by a Go or a NoGo trial), and (2) the type of sound (standard, deviant, silence). ANOVAs were carried out for each dependent variable, followed by theoretically-driven t-tests (Greenhouse-Geisser corrections were used when the sphericity assumption was violated). Effect sizes are reported as partial eta-square values for F tests, and as Cohen’s d_az_ for within-participant comparisons [75]. In addition to frequentist statistics, we also report the Bayes Factor (BF_10_) to assess the credibility of the experimental hypothesis relative to that of the null hypothesis given the data. Values below 1/3 are considered as substantial to strong support for the null effect, while values above 3 are regarded as substantial to strong supporting the presence of an effect [76,77]. Since past work has shown that deviant sounds yield some residual distraction in the next standard trial [11,41,78–85], and in line with past work controlling for this contamination effect [86–91], post-deviant standard trials were excluded from the analysis.

In line with previous work using the digit categorization task, overall performance was good (M = .89, SD = .05). The proportion of correct responses (see Table 1) was analyzed using a 2 (PostGo vs PostNoGo) x 3 (standard, deviant, silence) ANOVA for repeated measures. The main effects of trial type and sound condition were not significant; *F*(1,31) = 0.090, *MSE* = 0.006, *p* = .766, $\eta_{p}^{2}$ = 0.003, *BF*_*10*_ = 0.165, and *F*(2,62) = 0.345, *MSE* = 0.005, *p* = .710, $\eta_{p}^{2}$ = .011, *BF*_*10*_ = 0.070, respectively. These two factors interacted significantly, *F*(2,62) = 3.553, *MSE* = 0.006, *p* = .035, $\eta_{p}^{2}$ = .103, *BF*_*10*_ = 0.522. Comparisons between the standard condition and each of the other two sound conditions revealed no significant difference in PostGo trials: *t*(31) = 0.857, *p* = .398, *d*_*az*_ = 0.152 (95% CI: -0.198 to 0.499), *BF*_*10*_ = 0.265, for the deviant condition, and *t*(31) = -0.551, *p* = .585, *d*_*az*_ = -0.097 (95% CI: -0.444 to 0.251), *BF*_*10*_ = 0.217 for the silence condition. In the PostNoGo condition, no difference was found between the standard and silence conditions, *t*(31) = 0.981, *p* = .334, *d*_*az*_ = 0.173 (95% CI: -0.177 to 0.521), *BF*_*10*_ = 0.294, but the deviant condition yielded more correct responses than the standard condition, *t*(31) = -2.158, *p* = .039, *d*_*az*_ = -0.381 (95% CI: -.738 to -0.019), *BF*_*10*_ = 1.430. The difference between the deviant and silence conditions was modulated by the type of trial (PostGo vs PostNoGo): *F*(2,62) = 5.232, *MSE* = 0.008, *p* = .029, $\eta_{p}^{2}$ = .144, *BF*_*10*_ = 0.375. Overall, this pattern of data suggests a functional distinction between the effects of the deviant and silence conditions.

**Table 1:** Mean proportion of correct responses as a function of trial (postGo, PostNoGo) and sound (standard, deviant, silence) conditions. Values within parentheses represent one standard error of the mean.

	Standard	Deviant	Silence
PostGo	0.898 (0.010)	0.883 (0.018)	0.908 (0.016)
PostNoGo	0.889 (0.009)	0.917 (0.013)	0.872 (0.019)

**Fig 1:**
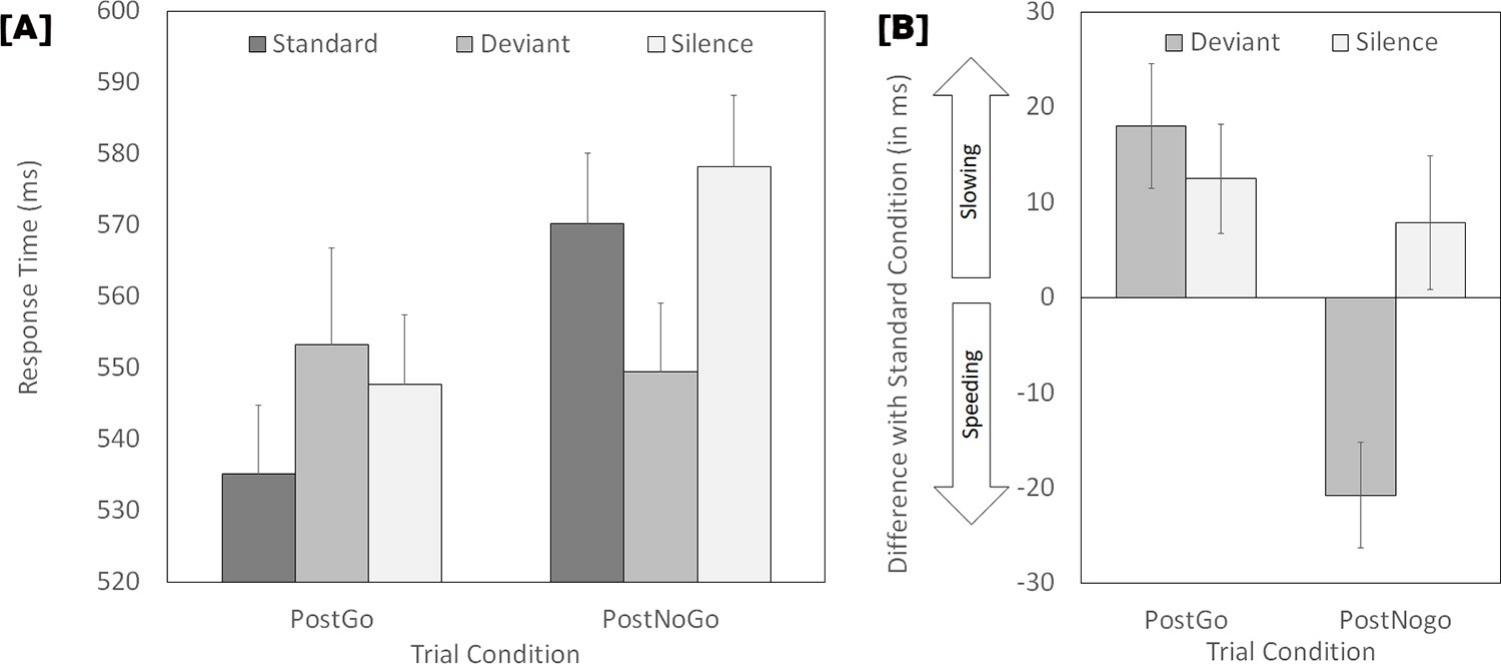
Response time performance. Panel A: Mean response times for correct responses as a function of the trial condition (PostGo, PostNoGo) and sound condition (Standard, Deviance, Silence). Panel B: Illustration of the differential effect of deviant sounds and unexpected silences in the PostGo and PostNoGo trials. The measure illustrated is the difference in response time relative to the standard condition. Error bars represent one standard error of the mean.

Mean response times for correct responses (see Fig 1, Panel A) were examined using a 2 (PostGo vs PostNoGo) x 3 (standard, deviant, silence) ANOVA for repeated measures. Participants were slower in the PostNoGo than in the PostGo condition, *F*(1,31) = 26.980, *MSE* = 756.368, *p* < .001, $\eta_{p}^{2}$ = .465, *BF*_*10*_ = 2717.574. The main effect of sound condition did not reach statistical significance, *F*(1.679,52.046) = 2.982, *MSE* = 1016.781, *p* = .068, $\eta_{p}^{2}$ = .088, *BF*_*10*_ = 0.462. However, a significant interaction was observed between these two factors, *F*(1.474,45.679) = 8.828, *MSE* = 1105.610, *p* = .002, $\eta_{p}^{2}$ = .222, *BF*_*10*_ = 112.327.

To analyze this interaction, we conducted two distinct ANOVAs. First, we carried out a 2 (trial type: PostGo vs PostNoGo) x 2 (sound condition: Standard vs Deviant) ANOVA aimed to test a replication of Parmentier’s (2016) findings (namely that, relative to the standard condition, deviant sounds slow responses in the PostGo condition while they shorten them in the PostNoGo condition). The main effect of trial type was significant: *F*(1,31) = 10.806, *MSE* = 728.160, *p* < .001, $\eta_{p}^{2}$ = .258, *BF*_*10*_ = 16.145. The main effect of the sound condition was not: *F*(1,31) = 0.101, *MSE* = 587.081,*p =* .753, $\eta_{p}^{2}$ = .003, *BF*_*10*_ = 0.190. However, the interaction between these factors was significant: *F*(1,31) = 19.120, *MSE* = 629.743, *p* < .001, $\eta_{p}^{2}$ = .381, *BF*_*10*_ = 407. Further examination confirmed that, in the PostGo condition, deviant sounds yield distraction relative to the standard condition: *t*(31) = 2.743, *p* = .010, *d*_*az*_ = 0.485 (95% CI: 0.115 to 0.848), *BF*_*10*_ = 4.402. In contrast, the effect was reversed in the PostNoGo condition: *t*(31) = -3.623, *p* < .001, *d*_*az*_ = -0.640 (95% CI: -1.017 to -0.255), *BF*_*10*_ = 31.750. Second, we carried out a (trial type: PostGo vs PostNoGo) x 2 (sound condition: Standard vs Silence) ANOVA to assess the effect of unexpected silences relative to the standard condition. The main effect of trial type was significant: *F*(1,31) = 44.663, *MSE* = 770.194, *p* < .001, $\eta_{p}^{2}$ = .590, *BF*_*10*_ = 1.893x10^7^. The main of sound condition was significant too: *F*(1,31) = 4.359, *MSE* = 760.372, *p =* .045, $\eta_{p}^{2}$ = .123, *BF*_*10*_ = 0.850, revealing overall longer response times in response to unexpected silences relative to the standard condition. Importantly, the interaction between these factors was not significant: *F*(1,31) = 0.325, *MSE* = 516.874, *p* = .573, $\eta_{p}^{2}$ = .010, *BF*_*10*_ = 0.290.

## Discussion

This study addressed a simple question: Do deviant sounds and unexpected silences yield behavioral distraction through the same mechanisms? In a cross-modal oddball task in which deviations from the standard sound consisted of a different tone or the omission of the standard sound, performance was compared as a function of whether the previous trial involved a target stimulus and a response (Post-Go and Post-NoGo trials, respectively). In line with past findings [67], deviant sounds yielded faster responses than the standard sound in Post-Go trials, but the opposite pattern was observed in Post-NoGo trials. This is thought to reflect the fact that deviant sounds help disengage from the current action plan: Following action, deviant sounds capture attention and cause distraction whereas, following a NoGo trial, deviant sounds help counteract action inhibition, thereby speeding up the response. In contrast, the omission of the standard sound lengthened response times in both Post-Go and Post-NoGo trials. Hence, the behavioral aftermaths of deviant sound and unexpected silence appear, at least in part, functionally distinct. The silence resulting from the omission of the standard sound appears to yield the effect expected of the removal of a warning signal rather than, or more than, that of a deviant sound.

The speeding of responses following an unspecific warning has long been documented [63,65]. It has been previously argued that the sounds’ warning property contributes to shorten response times independently from post-NoGo slowing, the deviant sound’s disruption of action plans, and individual differences in response speed [67]. The present results are in line with this proposition and indicate that the omission of the standard sound amounts, at least in good part, to the removal of a warning signal. In other words, the absence of the standard sound is functionally distinct from the presentation of a stimulus violating sensory predictions. This suggests that behavioral deviance distraction may be strongest when sensory predictions are compared to a positive stimulus (actual sensory input) and not, or much less so, to a negative one (absence of sensory stimulation).

Interestingly, some researchers have suggested that MMN is not a unitary response either, but the upshot of different mechanisms for deviant sounds and omissions [61,92]. According to this view, tone repetition leads to stimulus-specific adaptation, resulting in relatively small, evoked responses. In contrast, deviant sounds activate non-adapted sensory cells which, relative to the cells responding to the standard sounds, produce larger evoked responses. The MMN response triggered by the omission of the standard sound may, in contrast, correspond to an oscillatory N1, a reverberation due to the entrainment of the stimulation of sensory cells with the prior repetition of the standard sound. Empirically, the two types of MMN exhibit different temporal dynamics [60] and characteristics with respect to the rate of presentation of the sounds [59]. Finally, in studies in which the sensory consequences of the participants’ actions (pressing a button) are manipulated to elicit a sound on most trials, the omission of the predicted sound yields a N1 response greater than that of the predicted sound [93] and smaller than that of an unexpected sound [57].

Hence, taken together, our results and the findings from MMN studies suggest that the functional similarities exhibited by the effects of deviant sound and sound omission may result from at least partially different mechanisms. This does not imply that the same mechanisms underpin behavioral and electrophysiological responses to sound omissions, however. While N1 may play a role in the generation of a MMN-like response to sound omissions [92] and N1 appears to relate to alertness [94,95], the link is too tenuous to suggest a link between N1 and the warning value of sounds in the cross-oddball task. Parallels between behavioral and electrophysiological effects are generally perilous and past findings suggest that they are not directly linked. Indeed, the first can vary in the absence of fluctuation of the latter [26,71], and vice versa [96–99].

The present results suggest that in conditions functionally similar to those of the classic version of the cross-modal oddball task (Go trials following Go trials), the effects yielded by deviant sounds and the omission of the standard sound are undistinguishable. This presents a straightforward methodological implication: studies investigating the impact of stimulus omission as a potential source of deviance should make the sound uninformative (probability of a target following a sound set to chance), and data should be analyzed as a function of trial type (post-Go vs post-NoGo). Without this distinction, equating longer responses to omitted standard stimuli to the effect of a deviant stimulus [52] is questionable.

Finally, we should indicate that our results do not exclude the possibility that omissions may affect response times through both the removal of the standard sound’s unspecific warning signal (to a large extent) and as a deviant event *per se* (to a smaller extent). Our results certainly provide evidence of the first effect but does not rule out the possibility of the second, which may have been masked and made difficult to detect by the first. The first effect may for example be measured in using a design in which standard and silent trials are equiprobable (50% each) and should be smaller than in a design like ours (where standard trials represent 80% of the trials and deviant and silent trials 10% each). It may also be possible to gauge the two effects by manipulating the extent to which silent trials are predictable (thereby enabling or disabling the surprise associated with these trials). Overall, the results from the present study suggest that, in the cross-modal oddball task, the unexpected omission of the standard sound and the deviant sound lengthen response times in the primary visual task through different mechanisms, or possibly through different balances between distinct effects such the use of the sound’s warning value to prepare for action, the time penalty reflecting the involuntary orienting of attention to (and reorientation from) the deviant sound, and effect of deviant sounds on the selection of task-set or sensory-motoric mappings). At any rate, our results show that deviant sounds and the omission of sound do not yield the same impact on behavioral performance.
